# Digital Immunoassays for Sensitive Quantification of Blood Biomarkers Using Solid-State Nanopores

**DOI:** 10.1021/acsnano.5c16690

**Published:** 2026-03-24

**Authors:** Liqun He, Breeana Elliott, Philipp Mensing, Kyle Briggs, Michel Godin, Jonathan Flax, James McGrath, Vincent Tabard-Cossa

**Affiliations:** † Department of Physics, 6363 University of Ottawa, Ottawa K1N 6N5, Canada; ‡ Department of Urology, 6927 University of Rochester Medical Center, Rochester, New York 14620, United States; § Department of Biomedical Engineering, University of Rochester, Rochester, New York 14627, United States

**Keywords:** solid-state nanopores, digital immunoassay, DNA nanotechnology, single-molecule counting, traumatic brain injury, neurodegenerative disease, glial fibrillary acidic protein

## Abstract

Digital immunoassays enable highly sensitive detection of biomolecules, offering absolute quantification rather than relying on bulk signal intensity. We adapt a digital immunoassay scheme for a nanopore sensor, a versatile platform for single-molecule counting. Current nanopore sensors have demonstrated great progress when counting nucleic acids but struggle with proteins due to variability in translocation behavior and limited recognition strategies. While recent advancements have highlighted the promise of nanopore platforms for protein studies, precise quantification remains a challenge. Here, building on previous work, we present a nanopore-based digital immunoassay that employs gold nanoparticle-mediated molecular amplification with a single-molecule readout. This approach translates protein recognition into quantifiable DNA, enabling a precise digital assay. This assay employs a DNA NanoLock probe combined with a paramagnetic bead-based immunocapture, where the target proteins trigger a structural transformation of the NanoLock, converting their presence into a binary DNA-based signal. By incorporating AuNPs carrying hundreds of DNA proxy reporters, we effectively amplify the detectable signal by 2 orders of magnitude, significantly improving sensitivity. We validate the performance of this system by detecting the glial fibrillary acidic protein, a biomarker for traumatic brain injury and neurodegenerative diseases, in plasma samples and demonstrate high femtomolar-level sensitivity (∼40 pg/mL). Using the NanoLock probe, we further mitigate previous challenges, with reduced assay times (hours) and extended dynamic range (3-log). The self-calibrating nature of this digital approach offers robust, reproducible measurements across different nanopores, eliminating interdevice variability.

## Graphical abstract

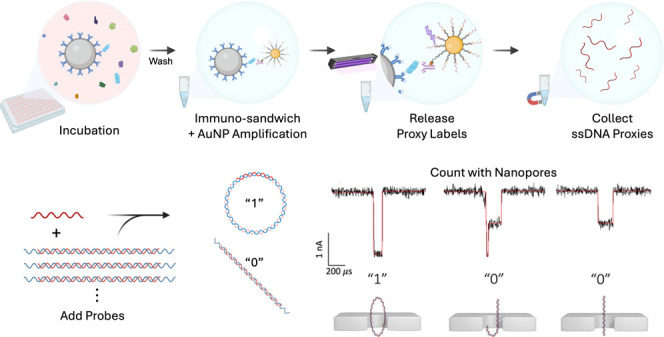

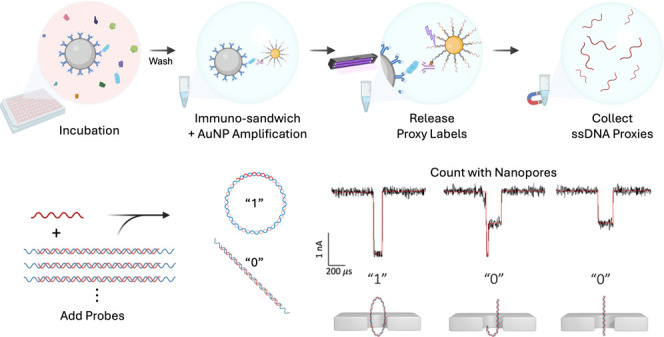

## Introduction

The pursuit of sensitive biomarker quantification is critical for early disease diagnosis and a timely intervention. Many medically relevant biomarkers are proteins, which may exist in very low abundances, often below the detection limit of conventional methods.^1,2^ While protein biomarkers can in principle provide a real-time snapshot of the health state of a biological system, accurate, point-of-care detection of protein biomarkers present in low concentrations poses significant analytical challenges.^3–5^ Traditional immunoassays based on ELISA,^6,7^ the mainstay of proteomic analysis for diagnostics, rely on an optical readout of antibody binding to a target and are limited to quantifying analytes in the pM range or higher. While recent work has pushed this limit,^8^ these advanced techniques remain firmly in research laboratories. To tap into the full potential of the plasma proteome, advancements in sensitivity and multiplexing that can be translated to the clinic are crucial. Currently only a small fraction of plasma proteins is used in routine diagnostics,^9^ pointing to the need for enhanced technologies that can provide deeper insights into the low-abundance spectrum of proteins.

Nanopores are single-molecule sensors that have been implemented in other contexts in a hand-held format.^10,11^ They have seen significant advancements in the past years, notably in the realm of nucleic acid detection and sequencing.^10–18^ This progress has been expanded to the characterization and fingerprinting of proteins,^19–26^ the analysis of carbohydrates, and the detection of viruses.^27–31^ Nanopores are becoming capable in vitro diagnostics tools, with their ability to rapidly and sensitively quantify disease biomarkers at the point of need.^32^ Solid-state nanopores, which are molecular-scale holes in robust synthetic membranes,^33–36^ have several attributes that make them strong candidates for diagnostics applications. Solid-state nanopores are highly customizable^37–39^ to accommodate various targets, are durable under a wide range of operating conditions,^39^ and their fabrication is compatible with integration into dense arrays within microfluidic architectures and electronic systems using wafer processing technologies.^40^ However, the journey toward diagnostic applications has been gradual, facing numerous obstacles.

Clinically relevant biomolecules, particularly proteins, are often incompatible with the conditions used in nanopore systems that yield optimal signal-to-noise ratios (high salt concentration). The transport properties of proteins through nanopores can be complex, and their rapid translocation challenges conventional electronics to resolve electrical signatures.^20,33,41,42^ Native solid-state nanopores also lack intrinsic specificity, requiring functionalization to recognize specific targets.^43–46^ Issues such as clogging and false positives are exacerbated when dealing with complex biological fluids.^47–50^ Additionally, variability in capture characteristics and transport properties between nanopores can hinder the standardization of results.^51^

Despite this, recent studies have made encouraging progress.^32,52–65^ DNA nanotechnology has been used to create carriers that can bind target proteins for subsequent readout by a nanopore,^66–71^ and recent work with bead-based immunoassays have pushed the limits of sensitivity for specific target proteins down to the femtomolar range with a single nanopore.^52^ While these approaches show potential, their sensitivity is still generally limited by the affinity of receptors^2^ to proteins and are often constrained by the size of the target relative to the receptor.

To address these challenges, we developed an alternative strategy that incorporates a DNA-based NanoLock probe for single-molecule solid-state nanopore detection. This approach leverages a structural transformation of DNA nanostructures, which shift from an open linear state (“0”) to a closed circular state (“1”) in response to the presence of a target protein. The transformation is mediated by proxy DNA reporters that are released upon target binding in a bead-based sandwich immunoassay,^2,52^ enabling a digital readout of biomarker concentration. Compared to our previous method,^52^ which relied on DNA nanostructures as presence/absence proxies, the NanoLock probe enhances assay efficiency by improving dynamic range, and reduces assay time by an order of magnitude. This digital nanopore immunoassay overcomes pore-to-pore variability in concentration measurements^51^ and achieves highly reproducible quantification of target proteins in complex biofluids. A schematic illustrating the idea is shown in Figure 1.

**Figure 1:**
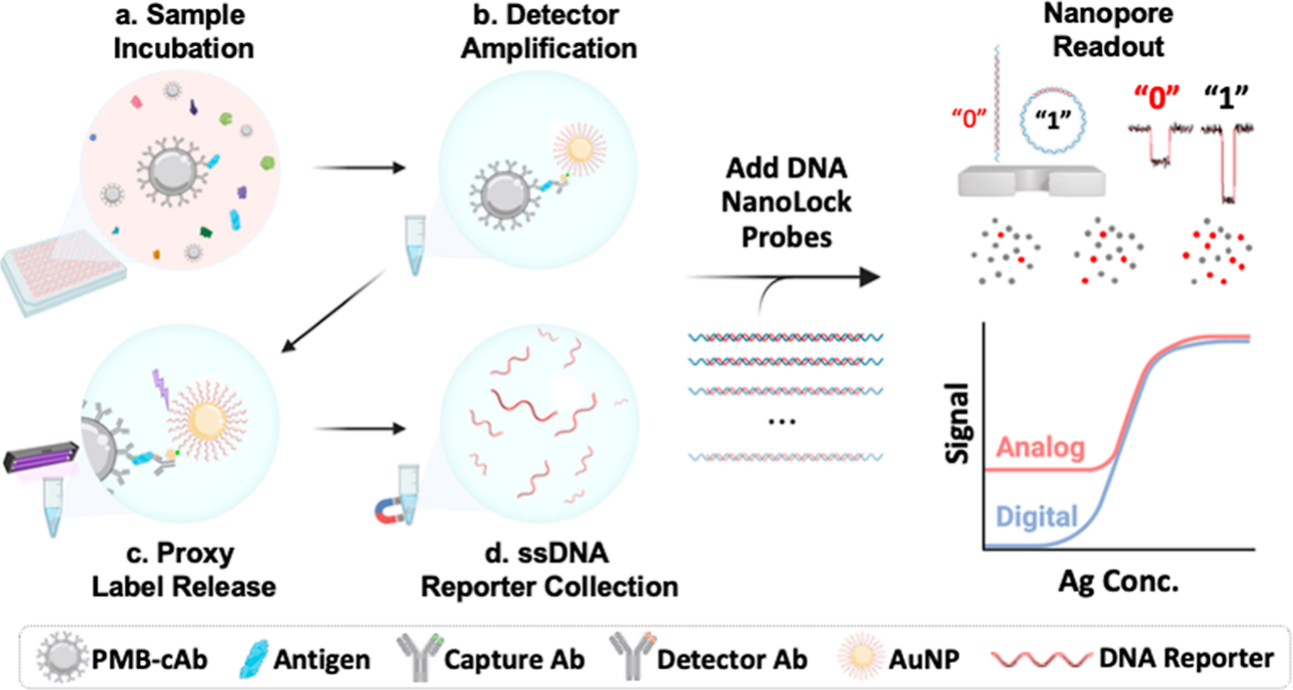
Schematic of nanopore digital immunoassay workflow. (a) Paramagnetic beads conjugated with antibodies (PMB-cAb) to capture specific target proteins in a blood plasma sample. Following washes, nonspecific molecules are efficiently removed. (b) The PMB-cAbs are incubated with detector antibodies with streptavidin attached. Unbound molecules are subsequently removed during next washes. The resulting immuno-complex is then amplified by attaching gold nanoparticles (AuNP) coated with hundreds of ssDNA reporters and biotinylated strands, proxy labels for the protein of interest. (c) The amplified immuno-complex is then washed and exposed to UV light in solution to release the proxy ssDNA reporter strands. (d) The PMBs are pelleted and immobilized, and the supernatant containing a concentration of reporters proportional to the initial target protein concentration is collected. Finally, a fixed concentration of the linear open-state DNA NanoLocks is added to the solution, and a proportion of the linear probes undergo shape transformation and form closed-state circular constructs for a given concentration of the reporter. The final solution is detected on a nanopore for digital readout by counting as “1” closed/circularized NanoLocks and “0” open/linear NanoLocks. Illustration was created with BioRender.

A key advantage of this approach over direct immunoassay approaches is the use of antibody-functionalized paramagnetic beads (PMBs) for target capture. The high local concentration of antibodies on the PMB surface amplifies the effective on-rate (*k*_on_) of target binding by a factor proportional to the number of antibodies per bead, effectively transforming each bead into a highly efficient capture entity; this dense antibody environment simultaneously reduces the effective off-rate (*k*_off_) by enabling the rapid recapture of dissociated targets. In this high-avidity complex, the target must avoid rebinding to any of the surrounding antibodies in order to fully escape, a process that is kinetically unfavorable. This enhancement enables femtomolar-level sensitivity, allowing for the detection of ultralow abundance biomarkers that are often below the limit of conventional immunoassays.^2,52^ Additionally, the PMB-based immunocapture scheme facilitates stringent wash steps, efficiently removing nonspecifically bound molecules and further improving assay specificity. The key advancement over our previous approach lies in the use of a single binding event for detection of proxy signals: whereas our previous approach involved binding two DNA origami structures together, the present approach simplifies this to locking a single linear DNA strand into a circular configuration, dramatically reducing the assay time and increasing the range of sensitivity compared to a two-step binding process.^52^ We also employ gold nanoparticles (AuNP) as an amplification complex by attaching multiple reporter molecules to each AuNP. Each AuNP carries approximately 200 ssDNA reporters as proxy labels, so each target-binding event yields a 200-fold signal amplification, significantly improving the downstream assay sensitivity.

In this work, we validate this strategy through the quantification of the Glial Fibrillary Acidic Protein (GFAP), a biomarker for Traumatic Brain Injury (TBI), an area of study that has received significant attention in recent years from academia and industry alike.^72–75^ Improving on our previous work, we now perform our assay with plasma samples, rich in proteins and biomolecules, which is a more challenging matrix, but which is closer to clinical relevance.^3–5,52^ To demonstrate the consistency of this method, we report results acquired on 11 nanopores, 72 experiments, and more than a million single-molecule events.

## Results and Discussion

To enable digital detection with a nanopore, we designed a DNA probe, called DNA NanoLock, as illustrated in Figure 2a (for more details, see the Methods Section and in Supporting Information Section S1, Figures S1 and S2). These NanoLocks, in their open-state, have a linear configuration. They are composed of a 400 nt long double-stranded region, with two 25 nt single-stranded overhangs at each end. The overhangs are complementary to respective halves of a specific ssDNA proxy reporter strand for the protein of interest. The introduction of such reporters, proportional to the target protein concentration, acts as a linker, initiating a transformation of the NanoLock, reconfiguring it into a circular shaped closed-state NanoLock. This probe design is optimized for nanopore detection; its distinct structural change provides a clear difference between the open and closed configurations in the nanopore electrical signal. The one-step binding of the reporter-probe design also enhances the binding kinetics to reduce the assay time: this scheme reaches equilibrium in 3 h (Figure 2j). Examples of single-molecule translocation events are listed in Figure 2b.

**Figure 2:**
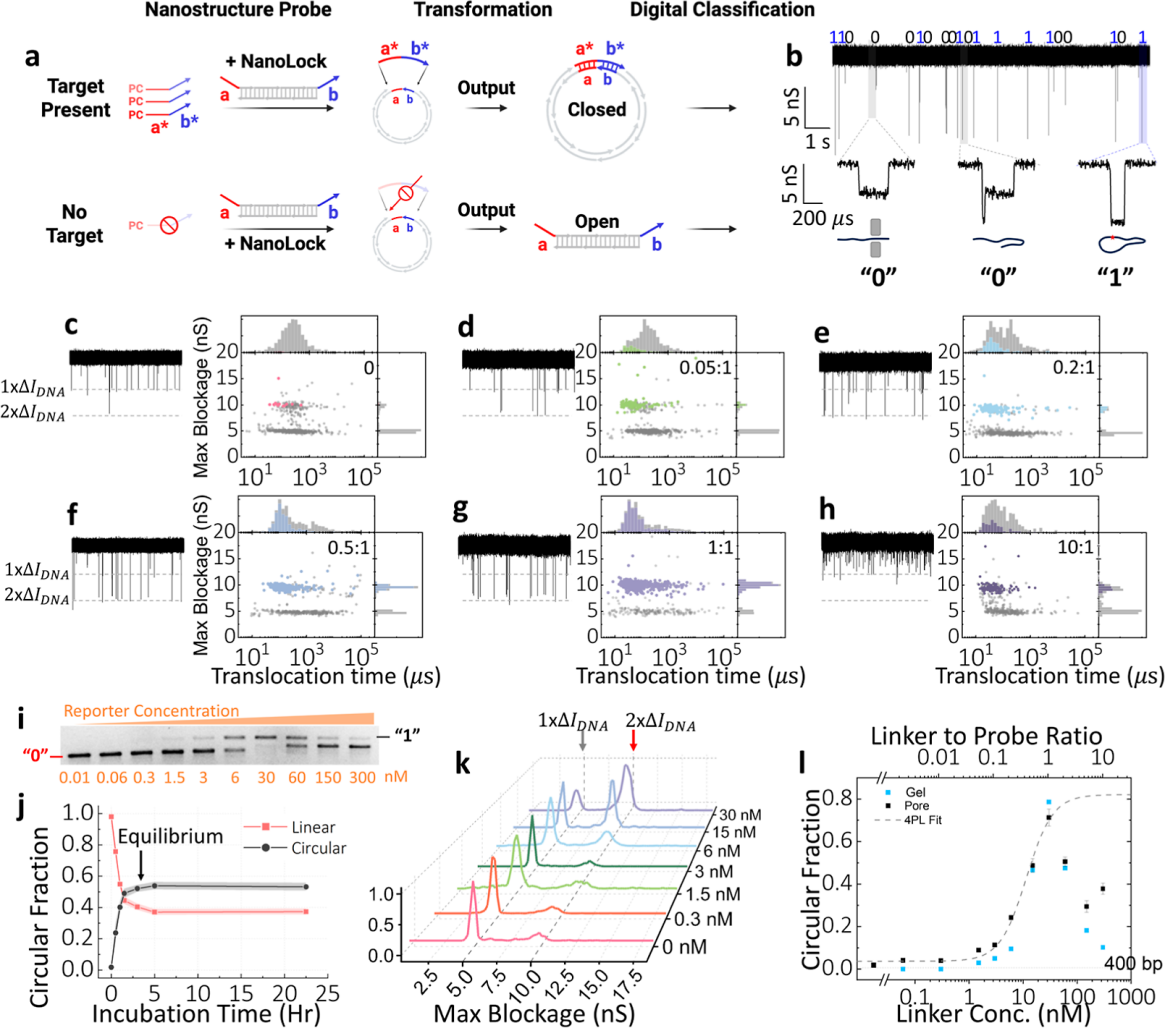
Nanopore translocation profiles of the DNA NanoLock open and closed states and its dose response. (a) Illustration of the transformation of the DNA NanoLock probes from open to closed states with the presence of an ssDNA reporter. (b) 10 s current trace of a mixture of NanoLock in its open and closed states, showing representative three types of translocations: linear single-file (Δ*I*_max_ = 1× dsDNA or 5 nS), linear folded (Δ*I*_max_ = 2× dsDNA or 10 nS followed by single-file), and circular (Δ*I*_max_ = 2× dsDNA or 10 nS), corresponding to “0”, “0”, and “1” in a digital scheme. (c–h) 10 s current traces and scatter plots of maximum blockage versus translocation time and their histograms at different reporter to NanoLock (probed fixed at 30 nM) ratios: 0:1, 0.05:1, 0.2:1, 0.5:1, 1:1, and 10:1, respectively. (i) Gel image of dose response of the DNA NanoLock, showing reporter concentrations of 0.01, 0.06, 0.3, 1.5, 3, 6, 30, 60, 150, 300 nM. (j) NanoLock closed state fraction versus incubation time for 0.4:1 reporter to probe ratio; the mixture reaches equilibrium in ∼3 h. (k) Histograms of maximum blockage of increasing reporter concentrations of 0, 0.3, 1.5, 3, 6, 15, and 30 nM, with the probes fixed at 30 nM. Blockage levels corresponding to 1× dsDNA ≈ 5 nS and 2× dsDNA ≈ 10 nS are indicated by black and red arrows. (l) Dose response for ssDNA reporter concentration ranging from 30 pM to 300 nM with a fixed NanoLock concentration of 30 nM. Nanopore experiments are performed in 3.2 M LiCl pH 8 at 150 mV using a 9.5 nm pore; a 200 kHz low-pass Bessel filter is applied for data analysis and current trace display. Gel electrophoresis (2% Agarose) is performed in 0.5× TBE buffer (at 100 V for 45 min).

Within this framework, the reporter strands serve as proxies for the target protein, while the use of multiple reporters per target effectively amplifies its concentration by 2 orders of magnitude (1 target: 1 AuNP: ∼200 reporter strands). This amplification factor was assessed by gel electrophoresis or with a nanopore sensor by using the DNA NanoLocks on a target with known concentration, as shown in Figure S4. The individual translocations of open-state NanoLocks are recorded as a digital “0”, while translocations involving closed-state NanoLocks—locked probes linked by the proxy label—are recorded as a “1”. This process translates the physical electrical signals from the nanopore into a binary digital format, simplifying the readout into a series of zeros and ones. Figure S5 compares digital to analog schemes for concentration measurement. By using a fixed probe concentration, the ratio of closed state events to total probes can be calibrated and precisely report on the concentration of target proteins present in the sample, with unbound NanoLocks effectively serving as their own internal calibration standard. As expected from controlled counting,^51,76^ the use of relative counts of each population allows for calibration-free quantification; therefore eliminating the error from the inherent variability of nanopores (e.g., change in capture rate over time, pore growth over time), allowing for highly reproducible assay performance that is consistent between nanopores and over time. It is worth noting that any systematic loss in reporter strand recovery during assay steps will manifest as a consistent bias in the digital readout. In practice, if the proxy release efficiency deviates, it is expected to simply result in a shift in the fraction of “1” events. This can be accounted for by a one-time calibration or reference measurement.

### Nanopore Validation of DNA Nanostructure Probes

To improve assay performance over previous work,^52^ we have developed an alternative DNA probe, the DNA NanoLock. The NanoLock nanostructures in its open and closed states provide a characteristic electrical signature when translocating a nanopore, as illustrated in Figure 2a,b. Briefly, the NanoLocks are hybridized using 15 50 nt long DNA oligomers to form a linear molecule. There are two single-stranded overhangs on each end of the NanoLocks, a (red) and b (blue) domains. The reporter strand consists of two domains, a* and b*, complementary to a and b, respectively, which serve as a linker. When the reporter strand is present, the two ends on the NanoLock, a and b, are joined to form a circular, locked, configuration. In contrast, the NanoLocks retain their linear open configuration if there is no reporter present. It is worth noting that a molecule that translocates the pore in a folded configuration may look like a circular strand if it is folded precisely in the middle of the molecule, resulting in a false positive. This is relatively rare,^77^ and such errors are easily corrected through calibration curves. Figure 2b shows a typical 10 s nanopore current trace as well as individual translocation events as insets, showing that the two NanoLock states events are easily distinguished. We further classified the translocation events in three types:

1. Translocation of closed-state NanoLock through a nanopore, denoted as digital “1”. These events produce a uniform peak with a current blockage level of ∼10 nS, corresponding to 2× dsDNA as two strands pass through the pore simultaneously.
2. Single-file translocation of open-state NanoLock, denoted as digital “0”, in which the structures translocate through the nanopore in a single-file fashion. These events produce a current blockage of ∼5 nS, which corresponds to 1× DNA.
3. The folded translocation of open-state NanoLock, “0”, in which the molecule translocates partially folded. This produces a two-step current blockage, a deep blockage of ∼10 nS corresponding to the folded portion, followed by a shallower ∼5 nS blockage corresponding to single file passage. Partially folded translocations in the opposite sense are almost never observed in practice as they defy tension-propagation principles.^78–81^

400 bp dsDNA with a similar length to the NanoLock without sticky ends is an ideal candidate for establishing an absolute negative control. The 400 bp control and the blank (no reporter) control registered false positive rates of 0.6% and 0.8%, respectively, as shown in Figure S3. Conceptually, given the persistence length of dsDNA (∼150 bp) and the length of the NanoLock (400 bp), the open NanoLock is expected to most often be captured by an end and pass through the nanopore single file.^82^ Realistically, as previously studied, the capture process is complex,^76,82,83^ and NanoLock molecules will not always approach the pore mouth by an end and result in some partially folded (type 3) passage.^77^ Due to the finite bandwidth of our measurements, we often miss the initial linear portion of the translocation when the NanoLocks are captured near the midpoint of their contour length. Nonetheless, we demonstrate that our digital classification scheme accounts for these partially folded translocations and reliably identifies open and closed events, with a false positive rate <1%. We note that for the immunoassay scheme presented below, the nanopore measurements are performed on released DNA reporters in a sensing buffer, and the nanopore is not directly exposed to biofluids (e.g., plasma), so that the baseline event-classification false positives remain <1% under these conditions (Figure S3). The background later observed in plasma experiments is attributed primarily to upstream assay steps rather than to the nanopore readout itself.

As a demonstration of our sensing scheme using this DNA NanoLock probe, we profiled the nanopore signature of these molecular structures and validated their dose response. We first assessed the response of the nanopore sensor using known concentrations of reporter strands and probes. For this, we fixed the concentration of NanoLock probes at 30 nM and varied the concentration of the ssDNA reporter strand from 30 pM (ratio of 0.01:1, reporter-to-probe) to 300 nM (10:1). Figure 2c–h shows the scatter plots of the maximum blockage depth versus translocation time for all single-molecule events recorded for six reporter-to-probe ratios: blank, 0.05:1, 0.2:1, 0.5:1, 1:1, and 10:1. Figure 2j shows the approach to equilibrium, while Figure 2k illustrates the appearance of additional blockage levels as the concentration of the target increases. The fraction of the circular events produced by the closed NanoLock can then be calculated for each concentration and used to construct a dose response curve, as shown in Figure 2l. The corresponding gel images and analyses are shown in Figure S4. 400 bp dsDNA was run on the same pore prior to the NanoLocks experiments. The closed fraction of 400 bp translocation data is plotted in a dashed line. As expected, with increasing reporter strand concentration below the fixed probe concentration, we observed a linearly increasing fraction of events attributed to the passage of circular (closed-state) NanoLock structures, reaching a maximum at a ratio of reporter-to-probe of 1, before the relative number of closed probes linearly decreased again. This nonmonotonic response can be modeled, under the assumption of irreversible first-order binding kinetics of reporter strands to probes.^52,57^ If the reporter to probe ratio, *x*, is less than 1, every reporter strand that binds to one end of the probes will eventually be able to find the other end with which to bind, leading to one locked structure per linker strand (i.e., *f*_Closed_ = *x*), with some variation for thermal fluctuations. Note that the terms linker and reporter strands are used interchangeably throughout the text. On the other hand, if there are more reporter strands than probes (*x* > 1), probes will get capped and all available binding sites, a and b, will be occupied. The probability of capping occurring before joining the ends is proportional to the ratio of concentrations assuming that diffusion times are not rate-limiting.

Figure 2k shows an overall maximum blockage shift as the linker concentration increases and the histograms for 0, 0.3, 1.5, 3, 6, 15, and 30 nM are shown, with the NanoLock fixed at 30 nM. Black and red arrows indicate blockage levels of Δ*I*_1× *dsDNA*_ ≈ 5 nS and Δ*I*_2× *dsDNA*_ ≈ 10 nS, corresponding to 1× dsDNA and 2× dsDNA, respectively. As the concentration of the reporter rises, the 1× dsDNA population diminishes, reaching a minimum at a 1:1 ratio. This trend is in line with expectations for the NanoLock’s transformation into a closed state. Figure 2j shows the time-evolution of the formation of lock state molecules, plotted as a function of incubation time for the 0.4:1 reporter to probe ratio. The red curve and black curve represent open and closed fractions, respectively. As the reaction takes place, the lock state fraction increases until it eventually reaches equilibrium and the curve plateaus, while the open state fraction decreases in proportion. Practically, we quantify in the monotonic regime (*x* ≤ 1) samples that fall into the nonmonotonic high-ratio regime (*x* > 1) susceptible to the hook effect^84^ that are quantified via split/dilution to return the response to a uniquely interpretable calibrated range.

While the NanoLock-based digital readout achieves high precision, the system does not show absolute binary behavior at extreme linker-to-probe ratios. At a linker-to-probe ratio of 0, where no linkers are present to induce circularization, a small fraction of closed-state events is still detected. This is likely due to rare false positives, instances where an open-state probe translocates the nanopore in a centrally folded translocation that mimics a closed-state event, consistent with the behavior of 400 bp dsDNA molecules alone (Figure S3a). We note that we cannot also rule out effects that may arise from spontaneous misfolding of DNA nanostructures where transient secondary structures resemble the closed state. Additionally, intermolecular interactions between misassembled probes could lead to unintended hybridization, mimicking a circularized configuration.

At the opposite extreme, a linker-to-probe ratio of 1:1, where complete circularization is expected, does not yield 100% conversion to the closed state. This deviation is likely due to inherent structural and kinetic constraints in DNA hybridization. The persistence length of double-stranded DNA imposes a structural limitation, requiring significant bending for the two ends of the NanoLock to hybridize, which may not always occur efficiently. This length was chosen to avoid additional analytical complexity arising from folding states. Additionally, misassembly or incomplete probe formation could result in missing or improperly exposed overhangs, preventing full hybridization. Although optimization of the probe purification workflow and sequence design could improve yield, in most assay conditions, consistent batch-to-batch probe synthesis should ensure consistent quantification.

Another contributing factor is probe dimerization, where a NanoLock end hybridizes with another probe molecule rather than its intended linker, reducing the fraction of fully circularized structures. Finally, thermal fluctuations guarantee some small proportion of unbound structures under any conditions. In this work, these effects do not impact assay performance as all measurements are performed with standard curves as a proof-of-concept. The small deviations from the theoretical binary behavior remain consistent across experimental conditions, allowing for robust and reproducible quantification. Furthermore, achieving absolute 100% or 0% circularization is not a strict requirement; rather, the system’s reliable and predictable response enables accurate measurements.

### Comparing Digital to Analog Nanopore Measurements

We compared the NanoLock to an analog capture-rate readout, where both digital and analog analyses are applied to the same raw nanopore data set, isolating the impact of the analysis strategy to demonstrate the benefit of a self-calibrating digital scheme. In an analogue scheme, the absolute amount (i.e., concentration) of a reporter molecule can be quantified from capture rate data, if a calibration curve is known for a particular pore size and operating conditions (e.g., voltages, electrolyte conditions). The capture rate is obtained either by the interevent time distribution for a particular molecule or by the total number of an event type in a given recording time.^51^ Figures 3 and S5 show the results from such an analog readout. While Figure 3a shows that event rate correlates with concentration, pore-to-pore variability affects the accuracy of the results.^51^

**Figure 3:**
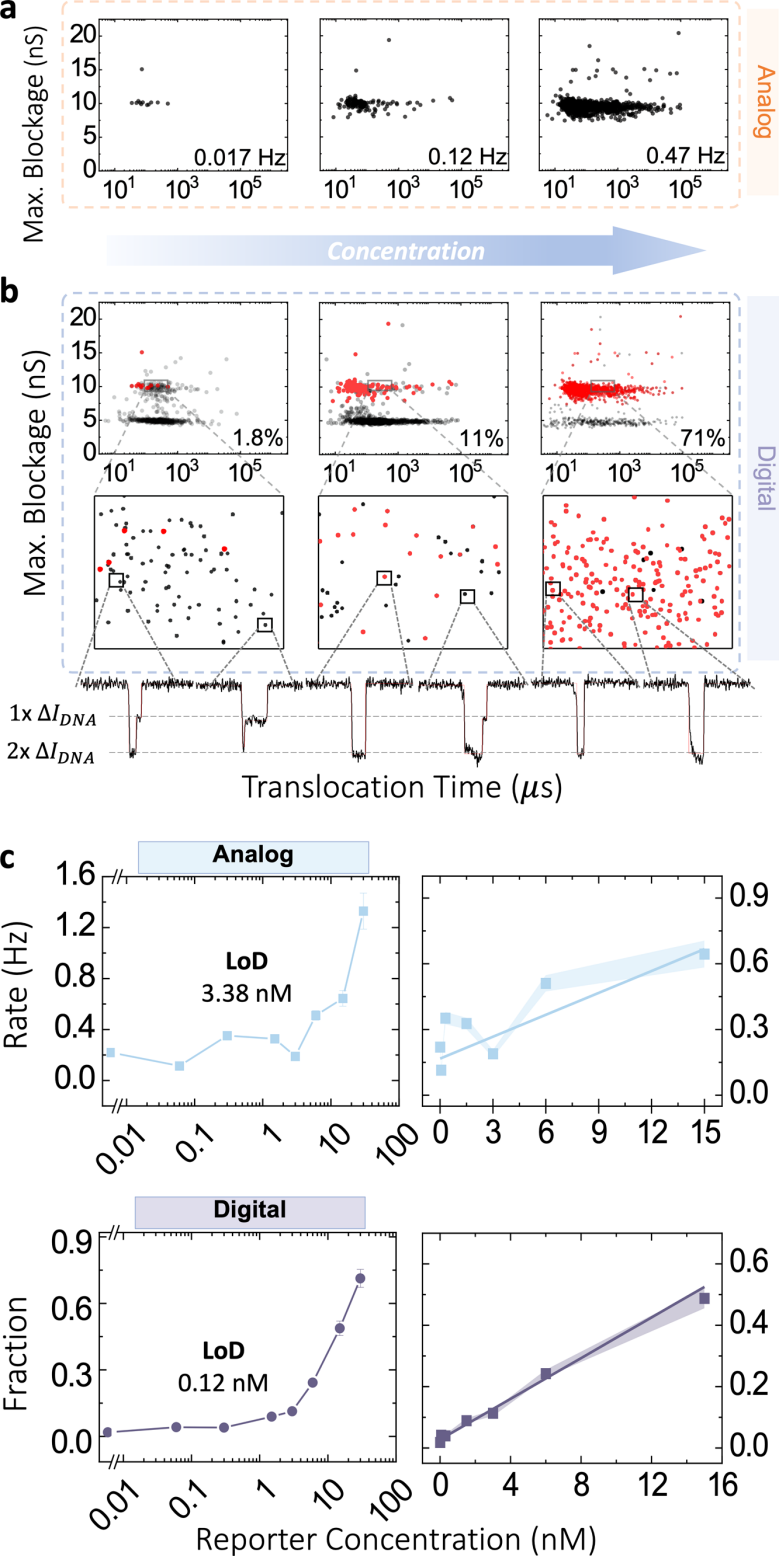
Nanopore digital and analog characterization of DNA nanostructure labels. (a) Nanopore analog detection of NanoLock open and closed state events. Scatter plots of maximum blockage versus translocation time for 0, 3, and 30 nM linker concentrations, showing capture rates of 0.017, 0.12, and 0.47 Hz, respectively. (b) Nanopore digital detection of open and closed NanoLocks using on single-molecule event shape analysis. Scatter plots of maximum blockage versus translocation time for 0, 3, and 30 nM linker concentrations. A rectangular region is zoomed in to show the single-molecule events of open (black) and closed (red) for the three concentrations. (c) Dose response for ssDNA reporter concentration ranging from 30 pM to 300 nM using analog and digital methods. Closed state events capture rate (analog) in the top row, and circular fraction (digital) in the bottom row, showing limit of detection (LoD) of 3.38 and 0.12 nM, respectively. Linear regions of the three methods are zoomed and displayed on the right. Experiments are performed in 3.2 M LiCl pH 8 at 150 mV using a 11 nm pore, a 200 kHz low-pass Bessel filter is applied for data analysis. The NanoLock probes are fixed at 30 nM for all experiments. LoD was calculated for each standard curve at 2.5 standard deviations above the blank.^2^

The analog mode yielded an LoD of 3.38 nM, as shown in Figure 3c (top row). In analog mode, the signal is defined as the capture rate of the “1” events (closed state events). However, at low target concentrations, the signal is buried in the noise from intra- and inter-experiment variations in the capture rate. In contrast, in the digital scheme, as shown in Figure 3b single-molecule events are counted to calculate a relative ratio, effectively eliminating pore-to-pore and experiment-to-experiment variabilities. Indeed, using digital classification, we observed an LoD of 0.12 nM that is > 10-fold lower than analog schemes (Figure 3c).

Besides improved sensitivity, this digital classification scheme shows promise toward multiplexed detection of many targets in parallel. Using DNA nanostructure labels which exhibit differentiable nanopore signatures,^85–88^ the population of events produced by each label can be identified and assigned, then individually calculated for “1” and “0” fractions to report on concentrations of analytes. Nanostructured DNA barcodes are promising candidates for this.^59,60,66^

### Nanopore Digital Immunoassay

To validate the performance of the proposed NanoLock digital assay with nanopore sensing, we quantified GFAP, a biomarker for TBI, in human plasma using the immunoassay workflow described in Figure 1. This strategy integrates paramagnetic bead-based immunocapture, gold nanoparticle (AuNP) signal amplification, and DNA NanoLock probes for a digital readout.

To deploy the DNA NanoLock in a sensitive immunoassay, we developed an amplification complex consisting of a AuNP decorated with releasable ssDNA linkers, enabling the amplification of the target protein concentration by 2 orders of magnitude before detection by the nanopore. We estimate that each 50 nm AuNP has a loading capacity of 200 copies of an ssDNA linker strand, based on the AuNP characterization detailed in Figures S6–S8. In this scheme, each target protein is translated to >200 copies of linker strands, which then bind to the ends of the DNA NanoLock to turn “open” NanoLocks into “closed” ones through hybridization, indicating the presence of the GFAP protein target, as shown in Figure 4a. We first ran the AuNP amplified immunoassay for GFAP concentrations of 0.5, 1, 2, 4, 8, 16, 33, and 66 pM in 4× diluted plasma. The nanopore current signatures are plotted in Figure 4b. Here, it is evident that more NanoLock probes are converted to the “closed” circular state, as the nanopore measurements reveal a shift from ∼5 to ∼10 nS in the maximum conductance with increasing concentrations of the target protein GFAP.

**Figure 4:**
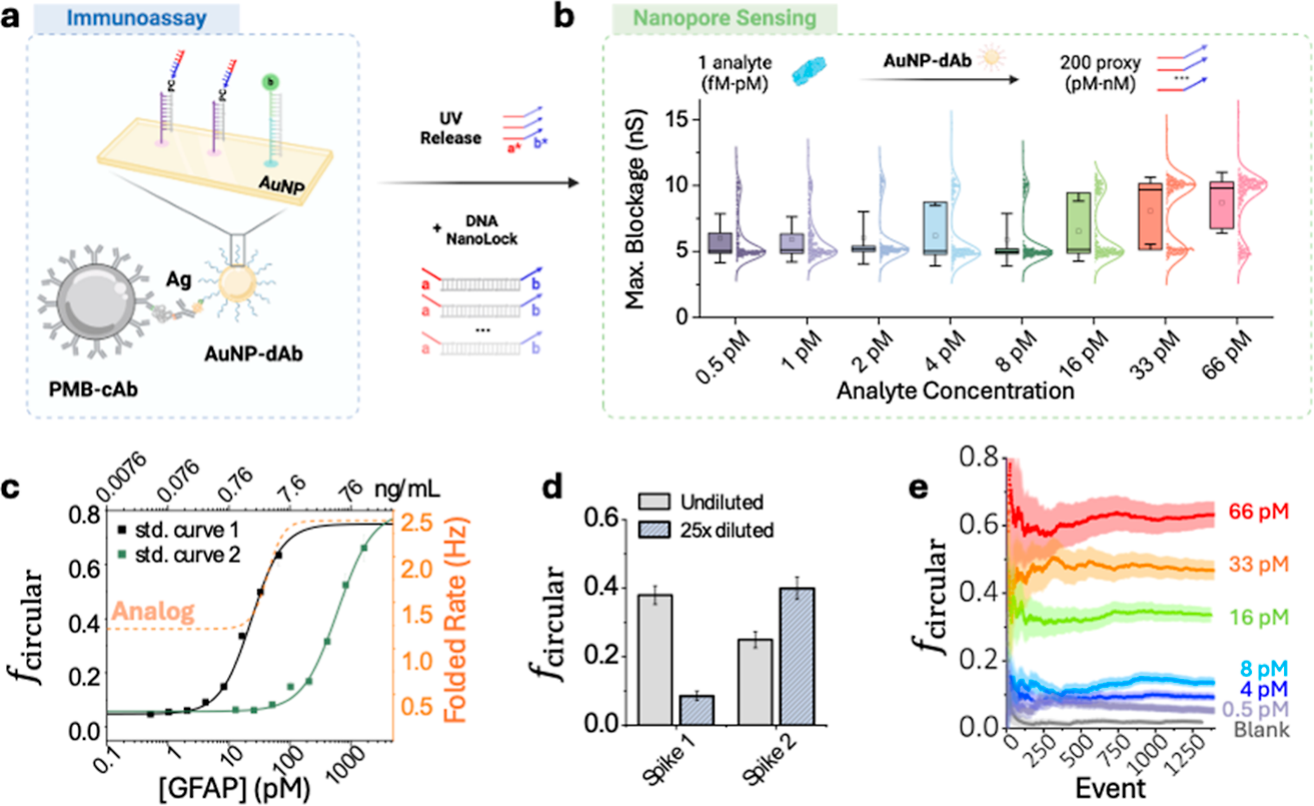
Nanopore amplification assay calibration curve and spike recovery. (a) Illustration of bead-based assay consisting of paramagnetic beads-capture antibody (PMB-cAb), antigen, and detection complex of DNA oligo-detector antibody decorated gold nanoparticles (AuNP-dAb). Streptavidin-coated paramagnetic beads (PMB-SA) and AuNP/biotinylated DNA oligo (AuNP) are used for validation of AuNP/oligo conjugation and oligo reporter release. (b) The complete assay immuno-sandwich is exposed to UV light to release DNA reporters, and NanoLock probes are added. The NanoLock reporter in its open state transforms into the locked state and sensed on a nanopore. Individual nanopore translocation events from an 8 pM GFAP (0.4 ng/mL) experiment and box plots of maximum blockage for GFAP assay concentrations of 0.51, 1.0, 2.1, 4.0, 8.2, 16.4, 32.9, and 65.8 pM. (c) Results of GFAP amplification assay, neat and diluted. Standard curve 1 (black) of GFAP concentrations, 0, 0.51, 1.0, 2.1, 4.0, 8.2, 16.4, 32.9, and 65.8 pM, for undiluted samples; and standard curve 2 (green) of GFAP concentrations, 12.8, 25.7, 51.4, 102.8, 205.6, 411.2, 822.4, and 1644.8 pM, for the diluted samples. The black and green lines indicate a 4PL fit of the standard curves for the two assay schemes, and the orange dashed line is the fit of the analog scheme using the capture rate. (d) Spike recovery for spike 1 (19.7 pM) and spike 2 (492 pM). Bar plots of spike 1 and spike 2 in two regimes, undiluted (gray, plain) and 25× diluted (light blue, sparse). (e) Time evolution of circular fraction as a function of an event, for GFAP concentrations ranging from 0.5 pM to 66 pM. The colored bands represent 1 standard deviation from the mean. Experiments are performed in 3.2 M LiCl at 200 mV using an 8 nm pore, all experiments are low-pass Bessel filtered at 200 kHz for analysis.

To ensure accurate quantification across a broad GFAP concentration range, each plasma sample was divided into two aliquots (as illustrated in Supporting Information Section S3): one left undiluted and the other diluted 25-fold in PBS containing 0.1% Tween-20. Both aliquots were independently measured and interpolated from two standard curves: Standard curve 1 covers low GFAP concentrations of 0.51, 1.0, 2.1, 4.0, 8.2, 16.4, 32.9, and 65.8 pM (black) for undiluted sample aliquots and standard curve 2 covering higher concentrations of 12.8, 25.7, 51.4, 102.8, 205.6, 411.2, 822.4, and 1644.8 pM (green) for the diluted aliquots. Each concentration from the standard curve 1 was sensed separately by a solid-state nanopore undiluted, while standard curve 2 was prepared at full concentration, then diluted 25-fold before nanopore analysis, as shown in Figures 4c and S9. The orange dashed line in Figure 4c indicates the standard curve using an analog (capture rate based) scheme. From the standard curves we can calculate an LoD of 865 fM for GFAP in plasma. This is comparable to the LoD determined for TSH in serum from our previous study. In the current configuration, the LoD is effectively set by the fixed NanoLock probe concentration; in principle, lowering the probe concentration would shift the working range downward, at the expense of longer hybridization and longer acquisition times.

To validate recovery, plasma samples were spiked with GFAP at two distinct concentrations of GFAP, 19.7 and 492 pM. Each spiked sample was processed under both conditions (undiluted and 25-fold diluted in PBS with 0.1% Tween-20), and concentrations were determined by extrapolation from the appropriate standard curve. Spike 1 (19.7 pM) showed circular fractions of 0.38 (from standard curve 1) and 0.086 (from standard curve 2). Spike 2 (492 pM) showed circular fractions of 0.25 (from standard curve 1) and 0.4 (from standard curve 2), as listed in Figure S10. For each spike, the higher fraction is picked and extrapolated in corresponding standard curves, with the resulting circular fractions for both spikes as well as the blank shown in Figure 4d. The 19.7 pM and 492 pM spikes show recoveries of 109 ± 8% and 110 ± 9% (Figure S10c), within the acceptable range of 80–120%.^89^ The time evolution of the circular “closed state” NanoLock events are plotted in Figure 4e, approximately 1000 events (∼10 min run time) are needed to be able to distinguish concentrations from 0.5–66 pM. Overall, this nanopore-based digital immunoassay demonstrates sensitivity down to the high fM range with >3 logs of dynamic range for GFAP in plasma.

### Study Limitations

This study is presented as a proof-of-concept for a NanoLock-enabled nanopore digital immunoassay. Multiplexed protein detection was not implemented; instead, we established a robust single-analyte workflow and a digital readout strategy that is, in principle, compatible with barcoded DNA proxy molecules for future multiplexing. In addition, as part of method development, performance was evaluated using spiked human plasma under controlled conditions; clinical patient cohorts and diagnostic performance metrics were not assessed here. Finally, although subfemtomolar operation is theoretically feasible, it has not yet been demonstrated in this study; reaching this regime would require higher throughput via parallel nanopore arrays, ultimately enabled by wafer-scale fabrication, which is not implemented in the current setup that uses the single nanopore readout.

## Conclusions

In summary, we have introduced a DNA NanoLock probe design that transitions from an “open” to a “closed” circular DNA form to report for the absence (“0”) or presence (“1”) of a protein target. Combined with a paramagnetic bead based immunocapture, and incorporating an amplification step relying on AuNPs carrying hundreds of DNA proxy reporters, we demonstrated the detection of GFAP, a biomarker for TBI, in plasma samples down to 865 fM with >3 logs of dynamic range. Similarly to our previous demonstration for TSH,^52^ the sensitivity of this nanopore-based digital immunoassay is limited by the lower bound of the concentration ratio of reporter strands to NanoLock probes that can be detected, which was determined to be slightly less than 1% (false positive rate), as shown in Figure S3. In principle, it can be adjusted to a lower concentration range by incubating with a lower NanoLock concentration. This comes at the cost of increasing the detection time if the measurement is performed on a single nanopore, as discussed in the Study Limitations section. Future developments should also consider strategies for enabling multiplexing with nanopores such as nanostructured DNA barcodes^60^ and validation with clinical samples.

## Methods

### Probe Assembly

The DNA NanoLock probes were assembled and purified as previously described.^52,90^ Briefly, the DNA NanoLock constructs were assembled from a set of 15 oligonucleotides (Integrated DNA Technologies), referred to here as oligos 1–15 (sequences are provided in Table S1). Equimolar amounts of each oligo (final concentration 0.3 μM) were combined in 1× TAEMg buffer (40 mM Tris, 20 mM acetic acid, 2 mM EDTA, 12.5 mM magnesium acetate, pH 8), heated to 95 °C for 5 min, then slowly cooled from 90 down to 60 °C at a rate of 0.4 °C/min, followed by a 0.03 °C/min ramp from 60 to 26 °C, and finally snap-cooled to 4 °C. All thermal steps were carried out in a MiniAmp Plus Thermal Cycler (ThermoFisher Scientific, #A37835). To verify assembly, 2% agarose gels in 0.5× TBE (pH 8.2) were used, and GeneRuler 1 kb plus DNA ladder (ThermoFisher Scientific, SM1331) served as a size reference. Bands were visualized with GelRed dye (Biotium, #41003).

The assembled NanoLock were purified on 5% Mini-PROTEAN TBE polyacrylamide gels (BioRad, 4565013). The appropriate bands were excised and then eluted using 3.5 kDa MWCO D-tube Dialyzer (Millipore Sigma, 71508 M). Probe concentrations were then quantified using a Take3 microvolume plate on an EPOC 2 spectrophotometer (BioTek, BTEPOCH2). For nanopore testing, the NanoLock probe concentration was fixed at 30 nM, with reporter strands ranging from 0.3 nM (0.01:1) to 600 nM (20:1) relative to the probe. Each mixture was incubated in 1× TAEMg for 3 h at ∼21 °C in a final volume of 35 μL.

### Assay Components

2.7 μm diameter carboxylated paramagnetic beads were conjugated with antihuman GFAP capture antibody (Quanterix, 102336). Conjugation was performed in accordance with SIMOA Homebrew Assay Development Kit procedures (Quanterix, 101354). 0.3 mg/mL capture antibody was incubated with 1.4 × 10^9^ beads.

For the gold nanoparticle (AuNP) amplification complex, 50 nm OligoREADY gold nanoparticles (Cytodiagnostics, OGC-50-2) were prepared following the protocols developed by Mirkin & co^91,92^ as well as manufacturer technical notes. The final solution was centrifuged at 2000*g* for 15 min, and the supernatant was removed. This wash step was repeated 3 times, and the final complex was resuspended in 200 μL of 1× PBS with 0.025% Tween 20.

For the assay standard curve, 7.2 × 10^7^ bead-capture antibody conjugates were mixed with varying amounts of GFAP control protein in 1× sample/detector diluent (Quanterix, 102336) for a total volume of 500 μL (volume used for all assay steps unless otherwise noted) and incubated for 1 h at room temperature (RT), 21 °C. To keep beads in suspension, tubes were placed on a 360° Multi-Functional Tube Rotator (VWR, PTR-35). All subsequent incubations and washes (>30 s) were performed on the rotator. Plasma samples from individuals were purchased from BioIVT. A 4× dilution was applied to the plasma sample to reduce matrix effects. For the amplification assay measurements, an identical procedure was followed: 10^7^ bead-capture antibody conjugates were mixed with varying amounts of control protein in 4× diluted plasma of total volume of 800 μL, and washes were done with 200 μL of 1× wash buffer instead. After initial incubation, three wash steps were performed by magnetically immobilizing the paramagnetic beads, removing the supernatant, and resuspending in 1× wash buffer 1 (Quanterix, 100486), with 5, 10, and 15 min intervals between each wash. Following washes, the immobilized beads were removed from the magnet and the pellet was resuspended in 500 μL of 1× sample/detector diluent containing 6 nM of biotinylated detection antibody with streptavidin and incubated for 30 min at RT. The gold nanoparticle amplification complex was added and incubated for 30 min. After incubation, to remove any excess of unbound reporter strands, three 30 s washes using 1× wash buffer were performed, followed by resuspension in 1× TAEMg with 0.1% Tween-20.

All samples were exposed to UV using a 3W LED flashlight (LIGHTFE, UV301D) at a distance of 1 cm for 20 min to cut the internal photocleavable linker and release the reporter strand from the immuno-sandwich. Reporter strands were recovered by magnetically immobilizing the remaining immuno-complex and recovering the supernatant with a pipet. To match the sensing range of the nanopore, a concentration step was performed to reduce the volume from 500 to 30 μL using an Amicon Ultra-0.5 Centrifugal Filter Unit (Millipore Sigma, UFC500396). The NanoLock probes were added to the assay supernatant at a final concentration of 30 nM each and incubated for 3 h.

### Assay Preparation

Aside from the use of a pseudo single-step binding to mitigate issues in our previous approach, the assay follows similar steps to that used previously: paramagnetic beads (PMBs) conjugated with capture antibodies (cAb) are used to bind targets and are then magnetically trapped to allow background and nonspecifically bound molecules to be eliminated during wash steps. Detector antibodies (dAb), which bind to different epitopes on the target protein, are introduced along with an amplification complex. This complex comprises a gold nanoparticle (AuNP) coated with hundreds of single-stranded DNA (ssDNA) reporters. These reporters have an internal photocleavable (PC) spacer, with a photolabile functional group that is cleavable by UV light. After equilibration, the complete immunocomplex (PMB-cAb, antigen, and dAb-AuNP) is magnetically immobilized and washed three times to remove excess components. The solution is then exposed to UV irradiation, photocleaving the reporter strands off the AuNPs, and releasing them into the supernatant, as described in our previous work.^52,90^ The supernatant, containing the ssDNA reporters in proportion to the target protein of interest, is recovered and mixed with a fixed concentration of DNA NanoLocks. These probes then transition from a linear shape to a circular shape through complementary DNA base pairing. The resulting solution is then added to the nanopore sensing buffered salt solution for analysis. In the current workflow, sample-to-readout time is ∼6 h, dominated by the 3 h NanoLock incubation, with nanopore acquisition on the order of minutes. The 3 h time was chosen as a conservative condition to ensure sufficient near-equilibrium circularization; shorter incubations may be feasible with optimization and appropriate calibration.

### Nanopore Fabrication

Nanopores were fabricated in 12 nm thick free-standing SiNx membranes (Norcada, NBPX5004Z) using controlled breakdown (CBD), which is described in detail elsewhere.^93,94^ CBD was performed in 1 M KCl buffered with 10 mM HEPES at pH 8 and pores were grown to 6 to 12 nm in 3.6 M LiCl buffered with 10 mM HEPES at pH 8 using moderate voltage conditioning. Prior to fabrication, the chips were cleaned using air plasma for 70 s and painted with a thin layer of PDMS to reduce high-frequency noise.

### Nanopore Sensing

The DNA nanostructures (in 1× TAEMg pH 8) were diluted to a final concentration of 3.2 M LiCl for nanopore sensing, where 3 μL of the nanostructure was added to 27 μL of 3.6 M LiCl buffered with 10 mM HEPES at pH 8. Linear 400 bp dsDNA fragments (ThermoFisher Scientific, SM1631) were always run prior to experiments involving DNA nanostructures as a molecular ruler to normalize away pore geometry variations during postprocessing. Samples were introduced to the *cis* side of the chip and a negative voltage was applied to the *cis* side with the *trans* side grounded. The ionic current recordings were performed in MATLAB 2013a (32 bit) using the VC100 current amplifier (Chimera Instruments) with a sampling frequency of 4.17 MHz and a bandwidth of 1 MHz and were subsequently software low-pass Bessel filtered as needed.

### Data Analysis

Nanopore signals were analyzed using a custom implementation of the CUSUM + algorithm,^95^ which is freely available online (https://www.github.com/shadowk29/CUSUM). A digital low-pass filter of 200 kHz was applied, unless otherwise specified. LoD was calculated for each standard curve at 2.5 standard deviations above blank.^2^ The fitted translocation events were plotted and further analyzed using Nanolyzer (version 0.1.41) from Northern Nanopore Instruments and Origin 2016 from OriginLab.

## Supplementary Material



## Data Availability

The raw data included this study are available from the corresponding author upon reasonable request. Liqun He, Breeana Elliott, Philipp Mensing, Kyle Briggs, Michel Godin, Jonathan Flax, James McGrath and Vincent Tabard-Cossa. Digital Immunoassay for Sensitive Quantification of Blood Biomarkers using Solid-State Nanopores. 2025, ChemRxiv Preprint, DOI: 10.26434/chemrxiv-2025-zth1z (accessed March 14, 2026). Code Availability Nanopore data analysis was done using in-house program available at https://www.github.com/shadowk29/CUSUM.

## Notes

Design and characterization of the DNA NanoLock; protocols for AuNP assembly and characterization; and additional GFAP immunoassay results (PDF)
