# Supplementary material for: Digital Immunoassays for Sensitive Quantification of Blood Biomarkers Using Solid-State Nanopores
Source: ACS Nano. 2026 Mar 24;20(13):10308–19. doi: 10.1021/acsnano.5c16690 (PMC13063814; doi:10.1021/acsnano.5c16690)
Supplement: Supplementary file 1 [file nn5c16690_si_001.pdf]

Supporting Information for

"Digital Immunoassay for Sensitive Quantification of Blood Biomarkers using Solid-State Nanopores"

Liqun He[1]*, Breeana Elliott[1], Philipp Mensing[1], Kyle Briggs[1], Michel Godin[1], Jonathan Flax[2], James McGrath[3]* and Vincent Tabard-Cossa[1]*

[1] Department of Physics, University of Ottawa, Ottawa, Canada, K1N 6N5;

[2] Department of Urology, University of Rochester Medical Center, Rochester, NY, USA, 14020;

[3] Biomedical Engineering, University of Rochester, Rochester, NY, USA, 14627.

*Correspondence: Vincent Tabard-Cossa (tcossa@uOttawa.ca)), James McGrath (james.mcgrath@rochester.edu), and Liqun He (liqunhe0215@gmail.com)

Table of Contents

Section S1. Design and Characterization of DNA NanoLock.. *2*

Section S2. Assembly and characterization of AuNP.. *8*

Section S3. Additional Nanopore Immunoassay Data...*11*

Section S1. Design and Characterization of DNA NanoLock

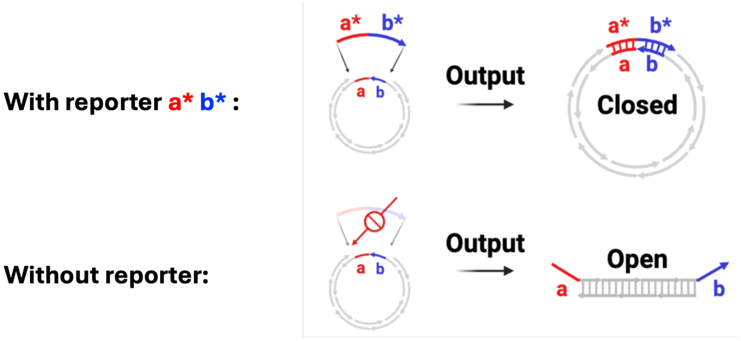

Figure S1. Design of DNA NanoLock. Oligos 1 - 15 form a linear "open" state NanoLock, the reporter strand consisting of domains a* and b* bind to overhangs a and b on the DNA NanoLock and transforms it into "closed" state.

The DNA NanoLock (**Figure S1**) is a programmable nanostructure composed of 15 single-stranded DNA (ssDNA) oligonucleotides (46–50 nt; sequences in **Table S1)** that self-assemble into a 400-bp linear duplex. This duplex feature two 25-nt single-stranded overhangs at its termini (domains a and b), which serve as hybridization sites for a complementary reporter strand (a*b*). Upon binding, the reporter bridges the overhangs, inducing a conformational shift from an open linear state ("0") to a closed circular state ("1"). The 350-bp duplex core provides structural rigidity, ensuring stability under nanopore sensing conditions (3.2 M LiCl, pH 8). This design shows the rapid binding kinetics highlighted in the main text **Figure 2** (reducing assay time to 3 hours).

Table S1: Sequences for the DNA NanoLock.

Name	Sequence	Length
1	CAACGAAGTCCTATTAGTAGAGCTGATGCCACCTCATCTGCTCGCGTCCG	**50**
2	TACAGATCTAAGTCGTACACAGGTTGCCTGAGTTGTGCGACGACGTCAGA	**50**
3	ATGGTCACGCTACGTCCAGCTACGGGCAGAATTGGGAATCAACTGTTACA	**50**
4	TGCTGTGAGACTTCCAGACACCGTCCTTTAGTTGCATATAAGCTTCATGT	**50**
5	CTACGTTCCTGGGTGACGAGCTAACCAGTGTGACAACTACAGGTACTCTC	**50**
6	CTCGATCATCAGTCGTAGTGTATCTCAGTGCTGGTGTTCAGATCTAGTGA	**50**
7	GGACGTTGTGAAGAGAACCTTCATCGGAGTAAGCTGTCTTGCCTCTATCG	**50**
8	GATGAAGGTTCTCTTCACAACGTCCTCACTAGATCTGAACACCAGCACTG	**50**
9	AGATACACTACGACTGATGATCGAGGAGAGTACCTGTAGTTGTCACACTG	**50**
10	GTTAGCTCGTCACCCAGGAACGTAGACATGAAGCTTATATGCAACTAAAG	**50**
11	GACGGTGTCTGGAAGTCTCACAGCATGTAACAGTTGATTCCCAATTCTGC	**50**
12	CCGTAGCTGGACGTAGCGTGACCATTCTGACGTCGTCGCACAACTCAGGC	**50**
13	AACCTGTGTACGACTTAGATCTGTACGGACGCGAGCAGATGAGGTGGCAT	**50**
14	TCGCTGAGGGAAGATGGCGAAACGATAGAGGCAAGACAGCTTACTCC	**47**
15	CAGCTCTACTAATAGGACTTCGTTGGTCACGAGTCACTATTTCGTC	**46**
Reporter	TTTCGCCATCTTCTCTCAGCGAGACGAAATAGTGACTCGTGAC	**43**
Capture Oligo	/5ThioMC6-D/CTATCCGGTGTGTATTCCAATGAGCAAACTGAGACTATGTCA	**42**
Spacer Oligo	ATTGGAATACACACCGGATAG	**21**
Reporter_PC	TTTCGCCATCTTCTCTCAGCGAGACGAAATAGTGACTCGTGAC/iSpPC/TGACATAGTCTCAGTTTGCTC	**64**
BiOligo	GTTGACAGTTCCGTGCTCAGCATCTCATACGACAGCATCCGAC/3deSBioTEG/	**43**
Bioligo Comp	GTCGGATGCTGTCGTATGAGATGCTGAGCACGGAACTGTCAACTGACATAGTCTCAGTTTGCTC	**64**

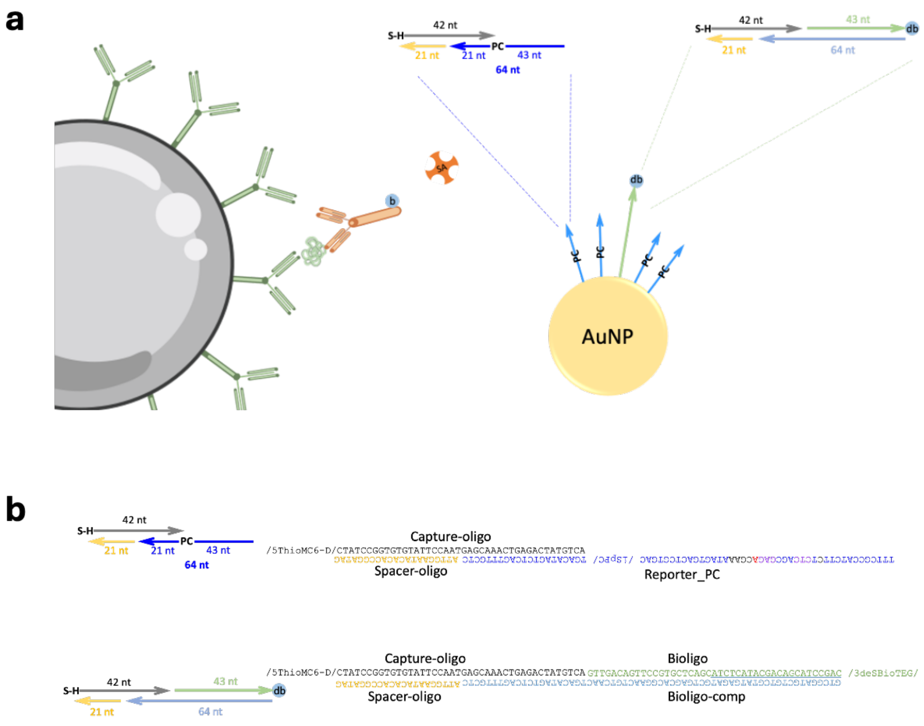

Figure S2. DNA oligo design for AuNP modification.

The gold nanoparticle AuNP amplification complex is functionalized with two distinct DNA constructs: (1) a thiolated 42-nt strand hybridized to a photocleavable reporter and a 21-nt spacer, forming a duplex that releases a 43-nt ssDNA reporter upon UV cleavage; and (2) a thiolated strand conjugated to a biotinylated DNA via spacer strands (in **Figure S2a**). Oligonucleotide designs and sequences are illustrated in **Figure S2**.

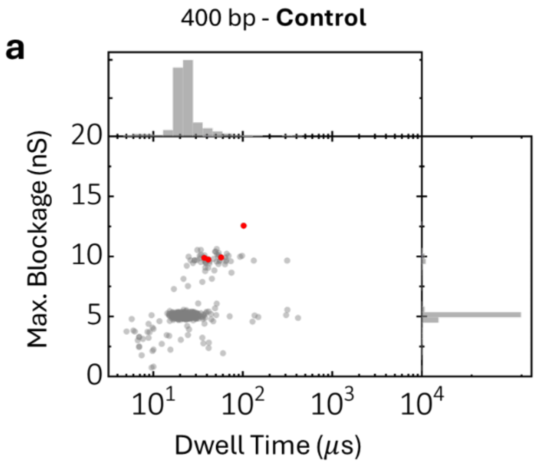

Figure S3. Nanopore translocation profiles of **a**) 400bp dsDNA and **b**) gel-purified NanoLock. The events classified as "closed state" or positive are plotted in red. Nanopore experiments were performed in 3.2M LiCl with an applied bias of -150 mV, using a 6.5 nm pore. Both samples were added at a final concentration of 30 nM. A 200 kHz low-pass Bessel filter is applied for data analysis.

Figure S3 shows the controls experiments, performed using a 6.5 nm solid-state nanopore under 3.2 M LiCl buffer (pH 8) with a −150 mV applied bias. The 400 bp dsDNA control (ThermoFisher, SM1701) exhibited single-file translocations ($\Delta I \approx 5$ nS, ~1× dsDNA diameter) with a 0.6% false-positive rate, validating the baseline for "0" events. The blank NanoLock (no reporter) showed similar behavior, with a 0.8% false-positive rate attributed to rare folded translocations (main text **Figure 2b**). Closed-state NanoLocks produced distinct ~10 nS blockages (2× dsDNA), confirming circularization.

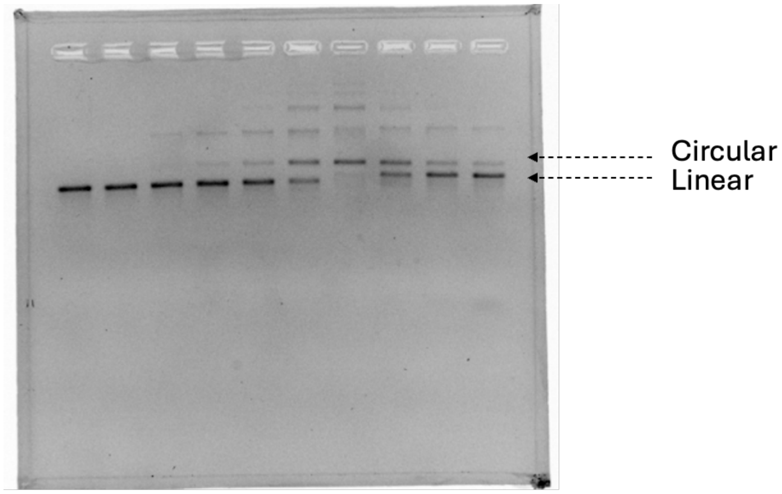

Figure S4. Gel image for main text figure 2, dose response of the DNA NanoLock. Gel image shows different reporter concentrations of 0.01, 0.06, 0.3, 1.5, 3, 6, 30, 60, 150, and 300 nM, respectively, with the NanoLock fixed at 30 nM. A 2% agarose gel (0.5× TBE buffer, 100 V, 45 min) was used to validate NanoLock transformation across reporter-to-probe ratios. SYBR Gold stained to reveal band shifts corresponding to closed-state formation.

As a demonstration of our sensing scheme using this DNA NanoLock probe, we profiled the nanopore signature of these molecular structures and validated their dose response. We first assessed the response of the nanopore sensor using known concentrations of reporter (linker) strand and probes. For this, we fixed the concentration of NanoLock probes at 30 nM and varied the concentration of the ssDNA reporter strand from 30 pM (ratio of 0.01:1, reporter-to-probe) to 150 nM (10:1). **Figure S4** shows different reporter concentrations of 0.01, 0.06, 0.3, 1.5, 3, 6, 30, 60, 150, and 300 nM, respectively, with the NanoLock fixed at 30 nM.

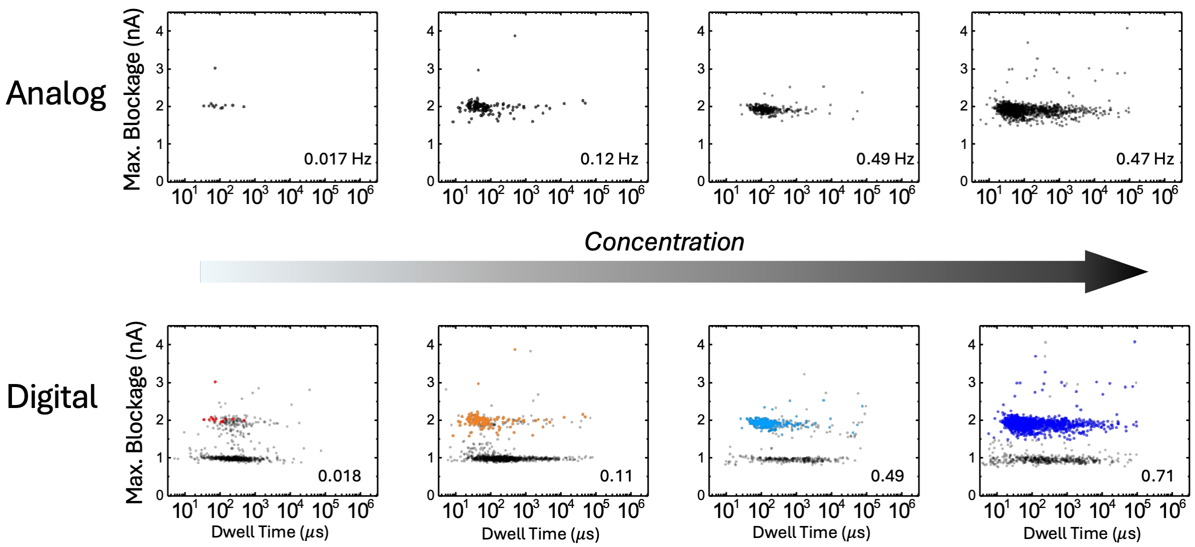

Figure S5. Comparison of digital and analog counting schemes using DNA NanoLock. Scatter plots are for reporter concentration of 0.01, 1.5, 15, and 30 nM, with the DNA NanoLock probes fixed at 30 nM. Experiments are performed in 3.2 M LiCl pH 8 at 150 mV using a 11 nm pore, a 200 kHz low-pass Bessel filter is applied for data analysis.

Figure S5 compares digital *versus* analog counting of the DNA NanoLock. For the comparison, digital counting scheme calculates a circular (closed state) fraction while the analog scheme is represented by the absolute capture rate of classified closed state events. The scatter plots are plotted for reporter strand of 0.01, 1.5, 15, and 30 nM, respectively, with the NanoLock fixed at 30 nM. Digital counting yielded circular fractions of 0.018, 0.11, 0.49, and 0.71, for the four reporter concentrations respectively. Analog readout showed capture rates of 0.017, 0.12, 0.49, and 0.47 Hz, respectively.

Section S2. Assembly and characterization of AuNP

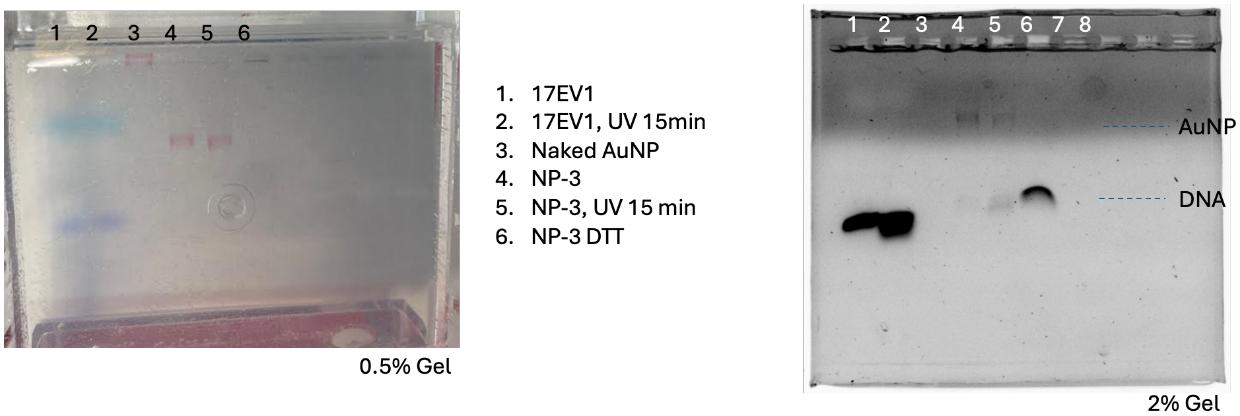

Figure S6. AuNP loading and release validation using agarose gel electrophoresis.

Agarose gel electrophoresis (0.5% gel, 80 V, 1 hr) validated AuNP DNA loading and UV-triggered release, as shown in **Figure S6**. Lanes included: (1) reporter DNA control, (2) UV-cleaved reporter, (3) bare AuNPs, (4) DNA-loaded AuNPs, (5) UV-treated AuNPs (released 43-nt reporters visible at ~43 nt marker), and (6) DTT-treated AuNPs (full release control).

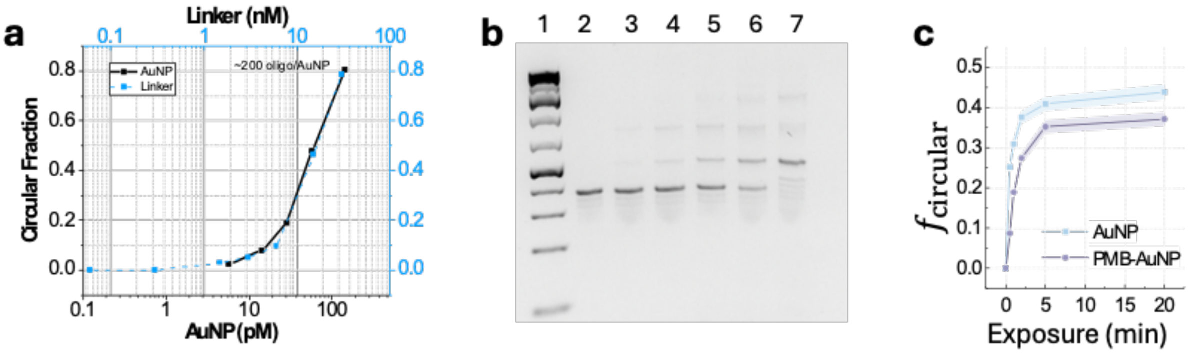

Figure S7. Characterization of AuNP, loading capacity and release efficiency using NanoLock. **a)** Nanopore characterization of loading capacity of AuNP using DNA NanoLock. **b)** Gel image of AuNP loading capacity, corresponding to nanopore experiments in a). **c)** Circular fraction *versus* UV exposure time, showing efficient of DNA reporter relaese from AuNP and PMB-AuNP in 5 min. 0.6 pmol of reporter oligo conjugated AuNP (OD = 10) is added to each experiment.

The loading capacity of AuNP are characterized using DNA NanoLock. Different amounts of AuNP were used in the experiment, the AuNP were UV treated, then the supernatant was incubated with DNA NanoLock. The responses of the circular "closed state" DNA NanoLock are plotted against and standard curve of reporter/linker DNA, as shown in **Figure S7a** and **S8b**. The loading capacity of AuNP is estimated to be > 200 copies of DNA per AuNP.

The release efficiency of the assembled AuNP were validated using UV exposure times from 5 minutes to 20 minutes (**Figure S7c**). To further validate the performance of AuNP on paramagnetic beads (PMB), we incubated same amounts of AuNP (contains biotinylated DNA oligo) on streptavidin coated PMB (ThermoFisher M270), and performed the same UV exposure. The supernatants were collected and incubated with the DNA NanoLock and ran on nanopores. Both just AuNP and PMB-AuNP show efficient UV release at 5-minute mark, the PMB-AuNP show smaller circular fraction.

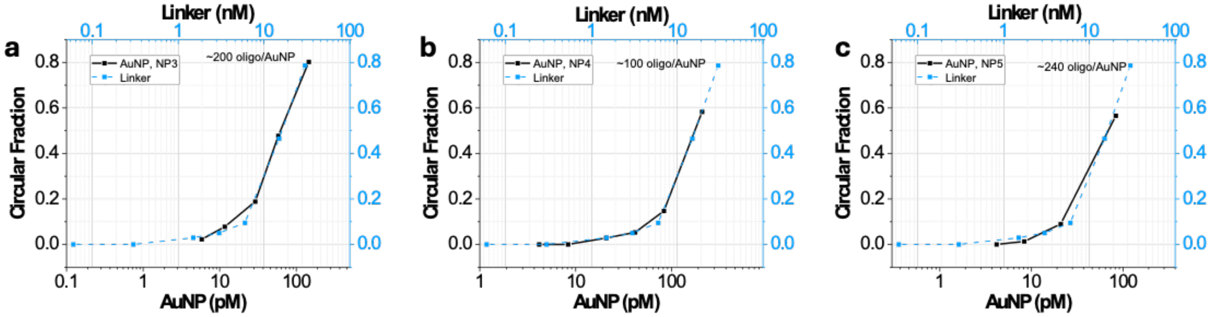

Figure S8. AuNP characterization and batch variation.

Three consecutive batches confirm loading capacity of 50 nm AuNP to be 100 – 240 copies of ssDNA per AuNP (**Figure S8**).

Section S3. Additional Nanopore Immunoassay Data

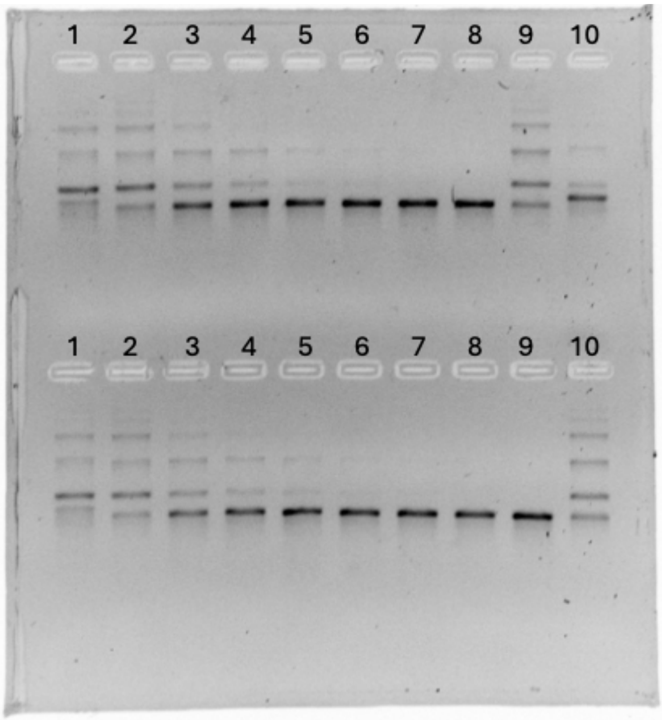

Figure S9. Corresponding gel image for main text figure 4.

Figure S9 shows gel electrophoresis results validating the AuNP amplification assay. Lanes 1–8 correspond to a standard curve of GFAP protein concentrations (ranging from 0.5 pM to 1645 pM) detected using the DNA NanoLock system. Lanes 9 and 10 show recovery of spiked samples (Spike 1 and Spike 2). Undiluted and 25x diluted samples are shown in the upper and lower gels, respectively. The undiluted gel shows 65.8, 32.9, 16.4, 8.2, 4.0, 2.1, 1.0, and 0.51 pM of GFAP, and diluted gel show 1644.8 pM, 822.4, 411.2, 205.6, 102.8, 51.4, 25.7, and 12.8 pM.

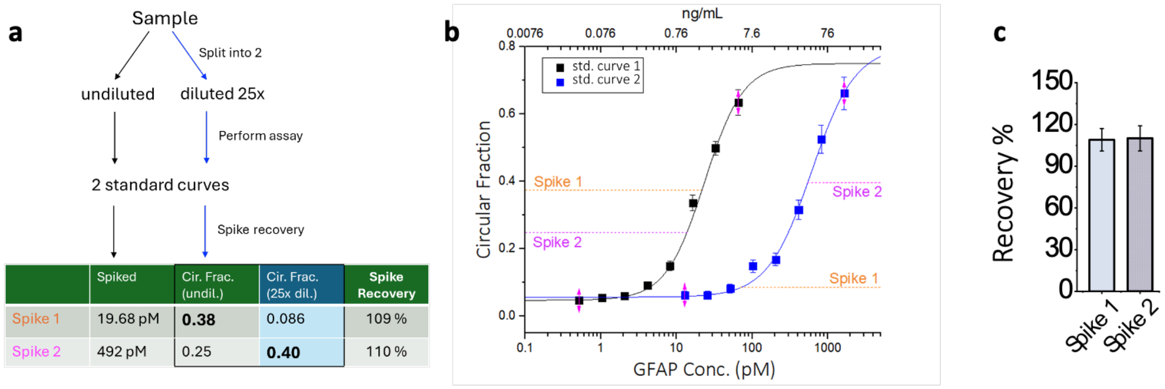

Figure S10. Corresponding nanopore amplification assay data for main text **Figure 4**. **a**) Sample split strategy for undiluted and diluted. **b**) Standard curves 1 (black) and 2 (blue) for undiluted and diluted samples. Undiluted and 25x diluted spike 1 (19.7 pM) and spike 2 (192 pM) circular fractions are labelled on both standard curves. **c**) Spike recovery for spike 1 (19.7 pM) and spike 2 (492 pM).
